# Dataset-centric evaluation of federated intrusion detection models in IoT networks

**DOI:** 10.1038/s41598-025-32567-w

**Published:** 2026-01-16

**Authors:** Muhammad Ahmad Bilal, Ihtesham Ul Islam, Sarmad Idrees, Muhammad Qasim, Muhammad Junaid Khan, Jaleed Khan

**Affiliations:** 1 Department of Computer Software Engineering, Military College of Signals, National University of Sciences and Technology, Islamabad, Pakistan; 2 Department of Information Security, Military College of Signals, National University of Sciences and Technology, Islamabad, Pakistan; 3 Department of Electrical Engineering, Military College of Signals, National University of Sciences and Technology, Islamabad, Pakistan; 4 Medical Sciences Division, University of Oxford, Oxford, Oxfordshire OX3 9DU UK

**Keywords:** Federated learning, Intrusion detection, IoT security, Dataset benchmarking, Attack diversity, Model generalizability, Engineering, Mathematics and computing

## Abstract

Intrusion detection systems (IDS) leveraging federated learning (FL) are increasingly deployed in Internet of Things (IoT) environments to address distributed data and privacy constraints. However, generalization remains unclear because most evaluations rely on a single dataset, which risks overfitting to site-specific traffic, label taxonomies, and non-IID client mixtures. This study provides a comprehensive dataset-centric evaluation of FL-based IDS across three contemporary IoT/IIoT datasets: Edge-IIoTset (2022), CIC-IoT2023, and TII-SSRC-23 (2023), that differ in devices, feature distributions, and attack families. We benchmark three FL aggregation algorithms (FedAvg, FedProx, FedNova) with two deep learning backbones (LSTM and Transformer) to assess detection accuracy, cross-environment generalizability, convergence behavior, and communication cost. Methodologically, we construct non-IID clients by device or application type, harmonize labels to a common family-level schema, align features to the intersection set, and evaluate three regimes: in-domain, cross-dataset, and a combined multi-dataset federation. Results show that federated models approach centralized performance in-domain, with macro-F1 up to 98% and accuracies in the 92–98% range. Transformers consistently exceed LSTM by $\approx$1–2% points in macro-F1 at comparable communication budgets. Cross-dataset tests expose substantial degradation, with up to 30 percentage-point macro-F1 loss when models face unseen environments, underscoring the need for diverse training coverage. Combined multi-dataset federation substantially restores transfer, yielding $\approx$90% macro-F1 across datasets in the harmonized family-level setting. Under heterogeneous clients, FedProx improves stability by reducing round-to-round variance, while FedNova achieves target accuracy in fewer rounds and lowers communication by $\approx$15–25% relative to FedAvg. These findings indicate a practical recipe for deployment: prioritize attack and environment diversity through combined-dataset FL, select Transformer backbones where feasible, and use FedProx or FedNova to stabilize training and reduce communication in bandwidth-constrained IoT settings.

## Introduction

The rapid growth of the Internet of Things (IoT) has dramatically expanded the attack surface of networks, making intrusion detection an essential defense mechanism for IoT environments^1^. Machine learning-based IDS have shown promise in detecting anomalies in IoT traffic; however, traditional centralized training approaches require aggregating potentially sensitive data from distributed IoT devices to a central server^2^. This raises privacy concerns and practical challenges given the volume and heterogeneity of IoT network data. Federated learning (FL) has emerged as a compelling alternative that enables collaborative model training across distributed devices without sharing raw data^3^. In FL, devices (clients) train a shared model on local data and periodically exchange model updates with a central aggregator (server) using algorithms like Federated Averaging (FedAvg). This paradigm preserves data privacy and can reduce communication of large raw datasets over the network.

**Fig. 1:**
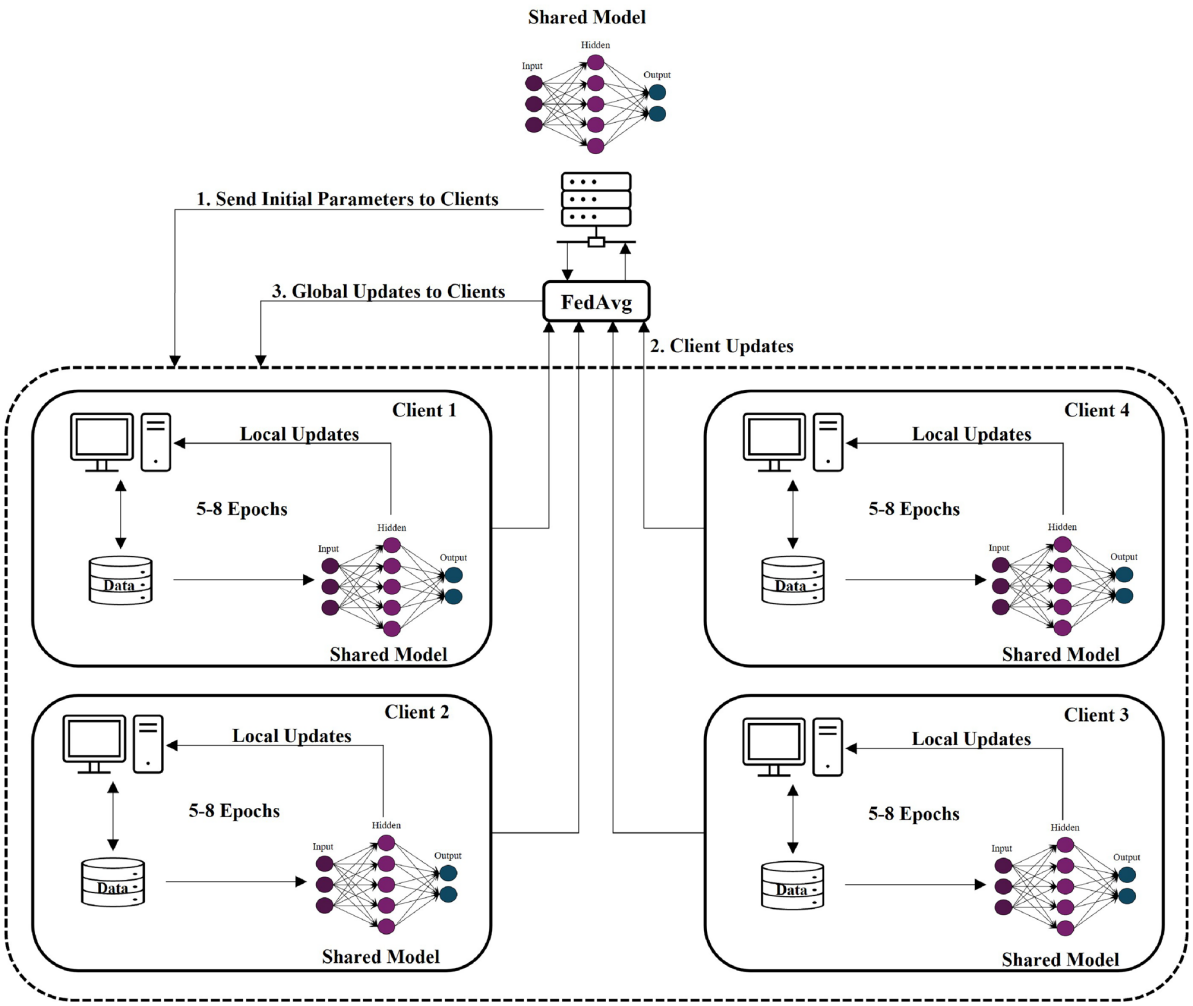
FL architecture for intrusion detection in IoT (illustration of FedAvg aggregation). Clients (edge devices or local servers) perform several epochs of local model updates on their partition of the data, then send updates to a central server which averages them (FedAvg) to produce a global model. This iterative process (1–3) continues for multiple communication rounds until convergence.

Recent studies have applied FL to IoT intrusion detection and shown that distributed training can achieve accuracy comparable to centralized IDS models^4^. Figure 1 illustrates a typical FL-based IDS architecture for IoT, where multiple client nodes (e.g. edge gateways or local servers) each train on local IoT network traffic and send model updates to a central server for aggregation using FedAvg. Through such iterative rounds, the global model learns to detect attacks collectively present across all clients. Prior works report that an FL IDS can be nearly as effective as a centralized model, while significantly reducing raw data transfer^5^. For example, in one study the federated model’s accuracy and F1-score approached that of training on all data centrally (within $\sim$1–2% difference)^4^.

Despite these advances, a crucial open question is how well FL-based IDS models generalize across different IoT environments and attack scenarios. Most existing evaluations train and test on the same dataset, risking overfitting to that dataset’s specific traffic patterns or attack types^6,7^. In reality, IoT deployments vary widely—from smart home networks to industrial IoT—and new attacks continually emerge. An IDS model trained on one dataset may underperform when faced with a different network context or novel attacks^8^. Researchers have thus highlighted the importance of attack diversity and dataset realism in developing robust IDS. The contribution of this paper is a systematic dataset-centric benchmarking of federated IDS models on multiple modern IoT/IIoT security datasets, to evaluate robustness and generalizability.

This work adopts a dataset-centric lens: we study generalization across distinct IoT/IIoT datasets and their differing class taxonomies, feature spaces, and distributions. We therefore operationalize “generalization” through three settings: (i) in-domain (single dataset), (ii) out-of-domain cross-dataset testing, and (iii) combined multi-dataset federated training with highly heterogeneous clients. Modeling temporal drift and incremental arrival of novel attacks is orthogonal to our goal and is left to future continual-FL studies.

Why a dataset-centric study? Each public IDS dataset has unique characteristics – background traffic profiles, sets of attack types (and their frequencies), feature representations, etc^9^. By comparing model performance per dataset and across datasets, we can identify how dataset attributes impact an FL model’s detection capabilities^10^. Our work leverages three recently released IoT/IIoT intrusion datasets that collectively cover a broad spectrum of attacks and network conditions:

- Edge-IIoTset (2022)—a comprehensive dataset of IoT and industrial IoT traffic introduced by Ferrag et. al^11^. This dataset emphasizes realistic IIoT scenarios and was designed to support both centralized and federated IDS research.
- CIC-IoT2023 (UNB CIC IoT Dataset 2023)—a large-scale IoT network intrusion dataset released by the Canadian Institute for Cybersecurity in 2023^12^. This dataset was collected in a realistic IoT lab environment to provide a benchmark for “plug-and-play” NIDS development.
- TII-SSRC-23 (2023)—a novel dataset by the Technology Innovation Institute (TII) that focuses on traffic diversity^13^. It was explicitly created to address the lack of variation in older datasets, providing enriched malicious samples and modern attack patterns (e.g., Mirai botnet traffic).

These datasets allow us to evaluate IDS models under different conditions: Edge-IIoTset combines IoT and IIoT with numerous attack families^11^, CIC-IoT2023 represents a large real-device network under coordinated attacks^12^, and TII-SSRC-23 offers highly diverse and fine-grained attack subtypes^13^. By training and testing FL models on each, and also testing models across datasets (to simulate deployment on unseen environments), we can assess model robustness and generalizability.

Architectural choices follow the data: we harmonize label spaces across datasets for combined training; align features to the intersection schema; and form clients by device/application groups to mirror realistic deployment. Evaluation emphasizes out-of-domain performance and communication-accuracy trade-offs, reflecting the premise that coverage of attack diversity is the main lever for transfer.

Furthermore, we incorporate two deep learning architectures widely used in sequence modeling and anomaly detection: an LSTM (Long Short-Term Memory) recurrent neural network and a Transformer encoder. LSTMs have been popular in IDS for modeling sequential packet/flow features and have shown strong results on IoT intrusion tasks^14^. Transformer-based models, with their attention mechanisms, have recently been explored for multi-class intrusion detection on the CIC-IoT2023 dataset and demonstrated improved accuracy by capturing complex feature interactions^15^. By evaluating both an LSTM and a Transformer in our experiments, we examine whether newer architectures yield benefits in an FL setting and if they generalize differently across datasets.

Our research contributions are:

- A comprehensive evaluation of FL-based IDS on three contemporary IoT/IIoT security datasets, with detailed analysis of how dataset characteristics (attack diversity, class imbalance, etc.) affect detection performance.
- Empirical comparison of three FL aggregation algorithms—FedAvg^16^, FedProx^17^, and FedNova^18^—in terms of detection metrics, convergence speed, and communication overhead. FedAvg is the standard baseline, FedProx introduces a proximal term to improve stability on heterogeneous data, and FedNova uses normalized averaging to address objective inconsistency when clients perform different amounts of local work.
- Investigation of model generalizability: we test models trained on one dataset against the others to quantify performance degradation on unseen distributions, and explore a federated multi-dataset training scenario to see if combining data from all sources yields a more universal IDS.

Throughout, we report multiple metrics (accuracy, precision, recall, F1-score, AUC) and include data in tables and figures to illustrate key findings. The results provide practical insights for researchers and practitioners on how an IDS might perform when deployed in new IoT environments and highlight the importance of diverse training data for robust intrusion detection.

The rest of this paper is organized as follows. Section 2 reviews related work on IoT IDS datasets and FL algorithms. Section 3 describes the datasets and summarizes their attack profiles. Section 4 details our methodology, including the FL setup, models, and metrics. Section 5 presents the experimental results, divided into per-dataset performance, cross-dataset evaluations, analysis of communication efficiency, and discusses the implications of these results and potential improvements. Finally, Section 6 concludes the paper and suggests future research directions.

## Related work

### IoT/IIoT intrusion datasets

There is a long history of public datasets for network intrusion detection, but many widely used ones (KDD’99, NSL-KDD, UNSW-NB15, CIC-IDS2017, etc.) have limitations for modern IoT contexts. Traditional datasets often lack IoT-specific traffic and suffer from skewed class distributions (overwhelming benign traffic with only a few outdated attack types)^19^. In recent years, researchers have developed new datasets tailored to IoT and IIoT scenarios. For example, the TON_IoT 2020 dataset^20^ integrated telemetry from IoT sensors with network data, and Bot-IoT (2018)^21^ included IoT botnet traffic, but these too had shortcomings in diversity or realistic device behavior. The Edge-IIoTset dataset introduced in 2022 stands out by covering multiple IoT application domains and attack categories, specifically aiming to support both centralized and FL research.^11^ emphasize that Edge-IIoTset better reflects IIoT environments (including industrial sensor networks) and provides a comprehensive benchmark for evaluating intrusion detection methods at the network edge. Likewise, CIC-IoT2023 was created to address the gap of real-device, large-scale IoT traffic – it includes 105 devices ranging from smart cameras to light bulbs, with attacks like ARP spoofing, DNS poisoning, various DDoS floods, web exploitations, and the Mirai malware. Jony and Arnob, documented all 33 attack scenarios in CIC-IoT2023 and provided both raw pcap and extracted flow feature sets to facilitate research^12^. The TII-SSRC-23 dataset (released in 2023) pushes the envelope further by augmenting malicious traffic diversity: it launched 26 unique attacks with many variations (parameter tweaks, different intensities, etc.), grouped into 8 high-level traffic types^13^. This dataset was explicitly motivated by the observation that public datasets over-represent benign traffic and have “a scarcity of diverse malicious traffic,” which limits IDS models’ generalization. By enriching the variety of intrusions (while still reflecting realistic traffic patterns), TII-SSRC-23 establishes new baselines for both supervised and unsupervised IDS techniques. In our work, we leverage these three state-of-the-art datasets as representative testbeds to evaluate federated IDS approaches, as they collectively cover an unprecedented range of IoT attack behaviors and network conditions.

### FL for IDS

FL was first introduced by Google in 2017 as a privacy-preserving distributed learning paradigm, and it has since been applied to various security domains including intrusion detection^22^. A number of recent studies examine FL for network IDS (NIDS), particularly in IoT settings where data is naturally distributed across devices or edge sites. For instance, Lu et. al^23^ used an FL approach on IDS data and found only minor accuracy loss compared to centralized training, demonstrating the viability of collaborative learning for security monitoring. Lazzarini et. al^24^ evaluated FL on the ToN_IoT and CIC-IDS2017 datasets using a simple neural network and FedAvg, confirming that a federated IDS could achieve around 97–99% accuracy in binary classification and high precision/recall close to the centralized model. They also experimented with alternative optimizers (FedAvgM, FedAdam) but observed FedAvg remained among the best in their scenario. Other works have proposed enhancements to FL for IDS: for example, research on aggregation algorithms has shown that FedAvg may struggle when client data are non-identically distributed (non-i.i.d.), which is common in intrusion detection (e.g., one client might see mostly one type of attack while another sees different attacks)^25^. The FedProx algorithm was developed to tackle such heterogeneity by adding a proximal term that keeps local model updates closer to the global model, preventing them from drifting too far due to local bias. Li et. al^17^ showed that FedProx yields more stable and accurate convergence than FedAvg in highly heterogeneous settings, improving test accuracy by up to 22% in some cases. We include FedProx in our comparison for precisely this reason – our federated scenarios involve heterogeneous attack distributions across clients. FedNova is another recent method which normalizes client updates by their number of local training steps, thereby eliminating objective inconsistency when clients perform different amounts of work^18^. This can occur if, say, one client has a larger dataset and does more epochs per round, inadvertently dominating the global update^26^. FedNova’s normalized averaging ensures the global model converges to a stationary point of the true objective (as if all data were considered uniformly). In an IDS context, if some clients generate more updates (e.g., a busy network segment vs. a quiet one), FedNova could improve fairness and convergence speed^27^. Prior work has analyzed FedNova under generic heterogeneity, but IoT-specific, dataset-aligned federation across multiple modern IDS corpora has not been systematically benchmarked. Our contribution is not a new optimizer or backbone but a dataset-centric evaluation protocol that spans single-dataset, cross-dataset, and combined multi-dataset regimes, where FedNova’s normalization materially changes convergence and communication in the presence of extreme client heterogeneity.

### Deep learning models for IDS

Deep neural networks, including recurrent and attention-based models, are now prevalent in intrusion detection research^28^. RNNs (especially LSTMs) can model temporal dependencies in network traffic flows or sequences of packets, useful for detecting slow or multi-step attacks^29^. Several prior works report high accuracy using LSTM-based classifiers on IoT malware or attack detection tasks. For example, a recent LSTM approach on CIC-IoT2023 data achieved over 99% binary classification accuracy and strong multiclass performance for major attack categories (DDoS, spoofing, etc.), demonstrating LSTM’s effectiveness in capturing IoT traffic patterns^30^. Transformers, with their self-attention mechanism, offer an alternative that can capture long-range feature interactions without recurrence. Tseng et. al^31^ applied a Transformer model to the CIC-IoT2023 dataset, reporting slightly improved F1-scores over CNN and LSTM baselines for multi-class intrusion detection. They leveraged a Transformer encoder (without the decoder, since it’s a classification task) to process flow-based feature sequences, noting the model’s ability to handle the large feature set (46 features) and complex decision boundaries in the 33-class classification. In our experiments, we use an LSTM model and a Transformer encoder model of roughly comparable scale (we ensure both have similar order of magnitude in trainable parameters) as the IDS classifiers. This allows us to observe if one architecture has an advantage in federated training or in dealing with diverse data^32^. We do not heavily optimize the architectures, as our focus is comparative and on the FL aspect; however, the Transformer model does incorporate multi-head attention layers and positional encoding suitable for tabular time-series input (we follow design ideas from)^33^. Both models are trained as multi-class classifiers to identify either the specific attack type or class label for each input sample.

Our work intersects these areas by applying advanced FL algorithms and deep models to modern IoT IDS datasets. The related work suggests that FL can maintain high detection performance and that algorithms like FedProx/FedNova may yield benefits under data heterogeneity. It also highlights that using multiple datasets can uncover generalization issues that single-dataset studies miss. Next, we describe the datasets in detail and how we partition them for federated evaluation.

**Table 1:** Summary of IoT/IIoT intrusion datasets used.

Dataset (year)	Scope & environment	Total samples	Attack classes	Example attack types
Edge-IIoTset (2022)	IoT & IIoT (industrial) testbed; mixed network protocols	$\sim$21 million flows	5 attack categories + 1 benign class	DoS/DDoS, info-gathering, injection attacks, man-in-the-middle, malware attacks
CIC-IoT2023 (2023)	Large IoT network (105 devices, real hardware)	$\sim$46 million flows	7 attack classes + 1 benign class	DDoS, DoS, Mirai, Spoofing, Recon, Web, BruteForce
TII-SSRC-23 (2023)	Emulated smart network with diverse traffic	$\sim$8.7 million flows	4 attack classes + 1 benign class	BruteForce, DoS, information gathering, Mirai

TII-SSRC-23 contains 26 labeled attack subtypes; for main-text multiclass evaluation we group them into four high-level families (DoS, BruteForce, Information Gathering, Mirai) plus benign, consistent with dataset documentation. Subtype-level analyses are provided in the supplement.

## IoT intrusion datasets and attack diversity

### Dataset overview

Table 1 provides a high-level summary of the three datasets used in our study. All datasets include a mix of benign (normal) and malicious traffic records with each malicious record labeled by a specific attack type. However, they differ in scale and attack taxonomy. Edge-IIoTset contains approximately 21 million according to its authors. It spans 5 attack categories and includes 41 features per flow (derived from packet-level data). In contrast, CIC-IoT2023 comprises around 46.7 million network flow records (extracted from extensive pcap logs covering 105 devices). It defines 7 top-level attack classes, each corresponding to several specific attacks (33 total). TII-SSRC-23 has an intermediate size (the dataset is $\sim$8.7 GB including raw PCAP and CSV features); it has approximately 8.7 million flows. Uniquely, TII-SSRC-23 labels attacks at a fine granularity of 26 distinct attack subtypes, though for analysis one can also group them into the 4 broader threat categories (DoS, brute-force, etc.) mentioned in Table 1. For consistency with Edge-IIoTset and CIC-IoT2023 class granularity, we report TII-SSRC-23 results at the family level (4 attack families + benign) in Tables 7 and 8; subtype-level metrics are discussed qualitatively and deferred to the supplement. All three datasets provide a rich playground to test IDS models: Edge-IIoTset and CIC-IoT2023 include various network attack vectors (network floods, scans, injection exploits), and TII-SSRC-23 adds multiple variations and Mirai botnet traffic.

**Fig. 2:**
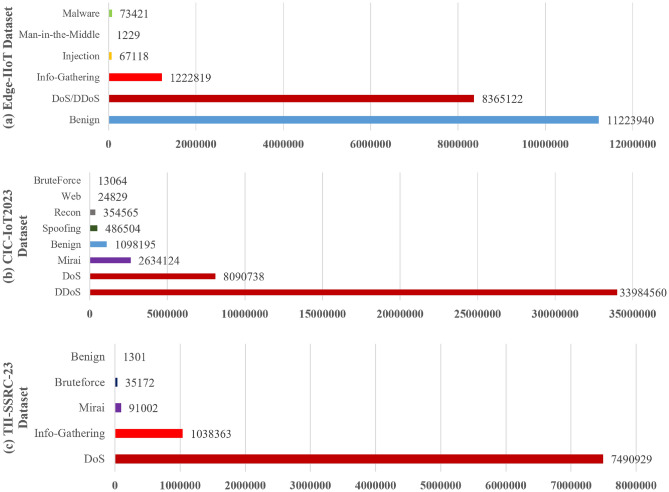
Distribution of records. (**a**) Edge-IIoTset dataset is highly imbalanced with several attack categories have relatively few samples, reflecting the realistic scenario where certain exploits are less frequent, (**b**) CIC-IoT2023 dataset contains several high-volume attack categories and all 33 specific attacks fall into these 7 classes; the figure aggregates at class-level for clarity, and (**c**) TII-SSRC-23 dataset grouped attacks into four high-level categories and contains many sub-types under each category (26 malicious sub-types total).

### Attack distribution and diversity

One major difference among the datasets lies in how the malicious traffic is distributed across attack types. This has implications for model training (class balance) and for evaluating how well a model can detect both frequent and rare attacks. Figure 2 visualize the attack distribution in each dataset (number of records per attack category).

- Edge-IIoTset^11^, as seen in Fig. 2a, exhibits an imbalanced distribution with a few attack types dominating. DoS/DDoS and information gathering attacks together make up the bulk of malicious traffic (in our illustration, roughly 8.3 million and 1.2 million records respectively), whereas specialized attacks like injection or malware are on the order of only 67–74k records. There is also a substantial benign portion—typically, benign traffic samples far outnumber any single attack type. The dataset’s creators acknowledge this imbalance but also stress that it reflects reality and that the diversity of types is more important for driving robust IDS development. For modeling, this means an IDS must cope with minority classes; techniques like class weighting or oversampling might be needed, but in FL settings not all clients may even see those rare classes, making it challenging (this is precisely where FedProx might help to not overfit one client’s majority class).
- CIC-IoT2023^12^ (Fig. 2b) has a more evenly spread attack distribution compared to Edge-IIoTset, albeit still skewed towards certain attack families. By design, each of the 7 categories contains at least one attack scenario; the DDoS category alone includes 11 different flooding attacks (ACK flood, UDP flood, Slowloris, etc.), which collectively produce a large number of malicious flows (we show $\sim$34 million, making it the largest category). The DoS category (distinct from DDoS in this dataset’s labeling) contributes another $\sim$8 million flows with 4 types of single-source floods. Notably, Mirai attacks account for a significant chunk ($\sim$2.6 million)—these include Mirai’s GRE IP and UDP flood behaviors. Meanwhile, brute force (only a dictionary SSH password attack) are very few. Reconnaissance attacks (port scans, ping sweeps, etc.) are also numerous ($\sim$354k). Overall, CIC-IoT2023 presents a large-scale, but somewhat balanced malicious dataset—multiple attack classes have substantial representation, which can facilitate training multi-class classifiers. However, the benign traffic in CIC-IoT2023 is also extremely large (tens of millions of flows), meaning that in a raw dataset the class ratio is still heavily tilted to normal. The dataset authors encourage evaluating both binary detection (malicious vs benign) and multi-class classification; in our study we focus on the multi-class aspect to stress-test models on fine-grained attack identification.
- TII-SSRC-23^13^ (Fig. 2c) aimed to introduce a wide variety of attacks, but not necessarily to balance them equally. From the figure, DoS attacks constitute the largest category of malicious data (e.g., various flooding attacks summing to $\sim$7.5 million flows). “Info Gathering” (reconnaissance scans, vulnerability probing) has around 1 million flows in our depiction. The Mirai botnet category (which could include Mirai’s scanning behavior, exploitation phase, and DDoS attacks launched by the botnet) is $\sim$91k. Brute force attacks (e.g., password guessing) appears small ($\sim$35k). The TII dataset emphasizes the breadth of sub-attacks. For instance, within DoS one might find several distinct vectors (HTTP flood, UDP flood, etc.), each maybe with a few thousand samples. While this provides an excellent test for fine-grained classification, it also means a model has to learn many classes with limited samples per class. The creators note that the benign traffic in TII-SSRC-23 is outnumbered by malicious (like most datasets), but they attempted to mitigate extreme imbalance by generating a relatively large set of malicious flows across those 26 subtypes.

### Common attack types

There is overlap in attack types across the datasets, which allows us to define some “common attacks” for comparative evaluation. All three datasets feature Denial of Service (DoS) attacks (including distributed DoS)—e.g., Edge-IIoTset and CIC have many forms of flooding; TII includes multiple DoS variants. All include scanning/reconnaissance activities (Edge’s “scanning”, CIC’s “Recon”, TII’s “info gathering”) and some form of brute-force password attack (Edge’s “password” attacks, CIC’s SSH dictionary attack, TII’s brute-force category). These three can be considered the core attack types present across all. Other attacks like Man-in-the-Middle (MITM) or spoofing appear in Edge and CIC (ARP spoofing is in CIC, and Edge lists MITM), but not explicitly in TII. Injection attacks (SQL injection, command injection) and XSS are present in Edge and CIC under web-based attacks, but not covered in TII. Backdoor/Malware attacks are represented in Edge (backdoor traffic, ransomware) and CIC (a “backdoor malware” scenario, plus Mirai which is malware)—for TII, the Mirai botnet category serves as the malware/backdoor representation. In our experiments we will sometimes focus on the common categories (DoS, scanning, brute-force) to compare performance uniformly. We also investigate the model’s ability to detect unseen attacks by training on one dataset and testing on another—e.g., how a model trained on CIC’s attacks performs on Edge’s unique ransomware traffic, or vice versa. This will shed light on attack generalizability.

## Methodology

**Table 2:** Federated data partitioning schemes.

Dataset	Clients	Partition strategy	Data distribution characteristics
Edge-IIoTset	6	By device/application type (each client has traffic from certain IoT/IIoT devices and associated attacks)	Moderate non-i.i.d.: some attack types appear only on specific clients (e.g., Client A might see mostly industrial-related attacks, Client B sees home IoT attacks).
CIC-IoT2023	10	By groups of IoT devices (approximately 10 devices per client)	Moderate non-i.i.d.: all attack classes present overall, but distribution varies: e.g., one client may contain more DDoS attacks if those targeted its device group heavily, another client might have more web attacks, etc
TII-SSRC-23	5	Random flow partition (each client gets a mix of all traffic types)	Nearly i.i.d.: each client receives a stratified sample of benign and all 26 attack subtypes. Minor statistical differences exist but largely balanced
Combined	3	Each client is an entire dataset (Client1 =Edge, Client2=CIC, Client3=TII)	Highly non-i.i.d.: completely different distributions per client (different feature scaling, attack mixtures, class definitions)

**Fig. 3:**
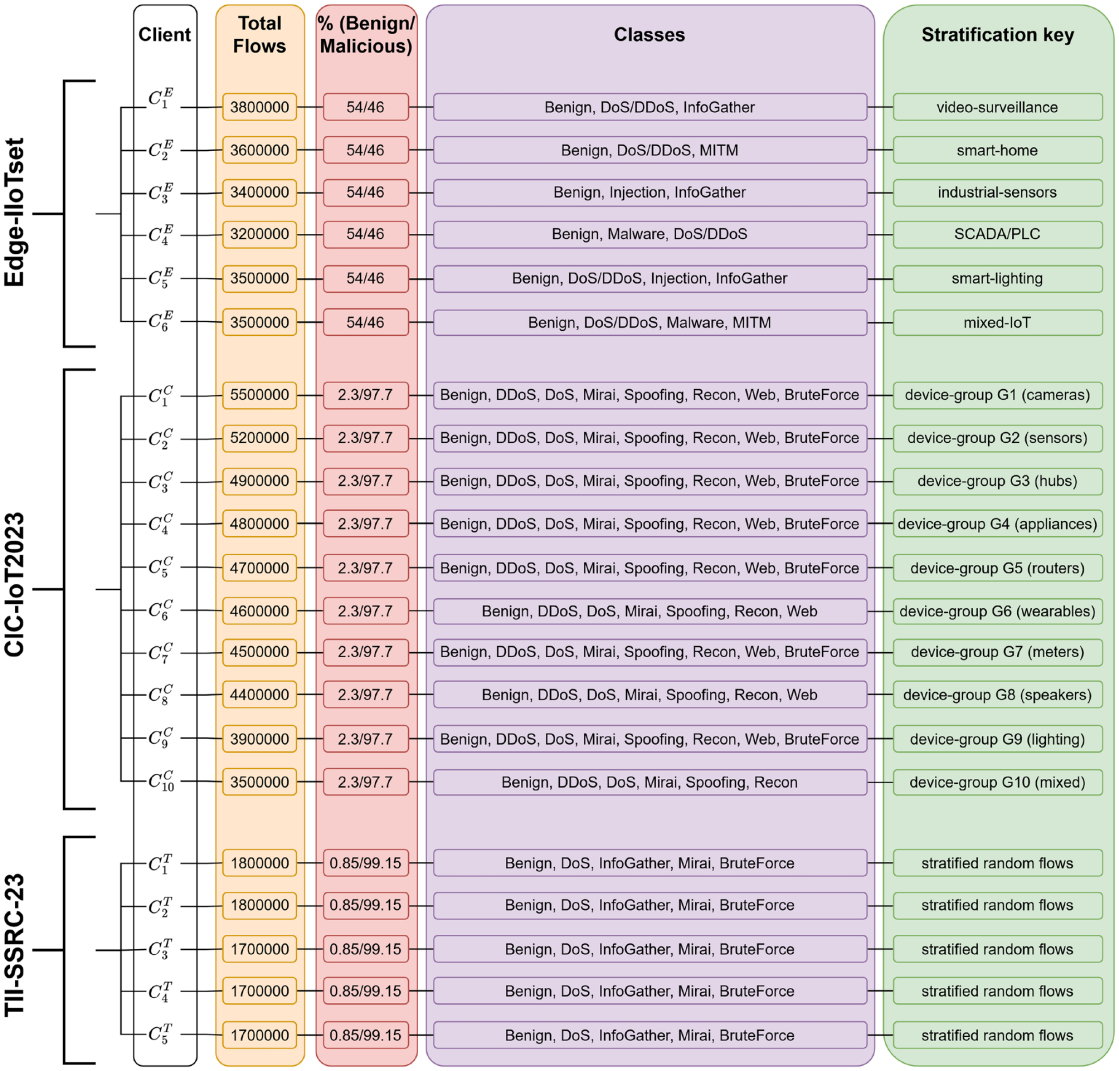
Client partitioning metadata and seeds. CIC-IoT2023 stratified by device-group, Edge-IIoTset by device or application type, TII-SSRC-23 by stratified random flow sampling. Global seed 42 with dataset seeds Edge=1729, CIC=2023, TII=1103. Exact client indices and split files are provided in the supplementary package. Clients are represented as $C_b ^a$, where a = (E $\rightarrow$ Edge-IIoT, C $\rightarrow$ CIC-IoT2023, T $\rightarrow$ TII-SSRC-23) and b is client ID.

### FL setup

We simulated FL separately for each dataset and also combined all datasets in a cross-dataset FL scenario. For single-dataset experiments, each dataset was split among multiple clients representing different organizations or network nodes (Table 2). For example, Edge-IIoTset was divided into 6 clients based on device types, creating a moderately non-i.i.d. distribution where each client had distinct attack profiles. CIC-IoT2023, larger in scale, was partitioned into 10 clients grouped by subsets of devices, resulting in somewhat more uniform distributions. TII-SSRC-23 was split into 5 clients with randomized but balanced traffic to simulate an i.i.d. setting. We map “environmental heterogeneity” to distributional shift across datasets and clients via (a) label-space mismatch and aggregation (Table 1), (b) feature alignment for multi-dataset training, and (c) client-level non-IID partitioning (Table 2). Temporal non-stationarity and continual incorporation of novel attacks are out of scope for this dataset-centric study. Client-level counts, benign versus attack proportions, class coverage, stratification keys, and seeds are summarized in Table 3.

Edge-IIoTset is partitioned into 6 clients by device/application; CIC-IoT2023 into 10 clients by device group; TII-SSRC-23 into 5 clients by stratified random flows. Per-client Total flows sum to Table 1 totals (Edge 21,000,000; CIC 46,000,000; TII 8,700,000); Benign/Attack shares per client match Fig. 2. Client metadata appear in Fig. 3 and are fixed across all runs.

Labels are harmonized to (Benign, DoS/DDoS, Recon/InfoGather, BruteForce). Features are aligned to the intersection of 40 numeric flow features with z-score normalization (fit on training split only); in cross-dataset tests the source scaler is applied to the target. No domain adaptation is applied in main results. The full feature intersection is listed in Table 3.

In the combined scenario, each client represented one entire dataset, forming a highly heterogeneous federation across different network environments. We aligned these datasets by selecting only common features and normalizing them to ensure consistency. The combined label space included all attack classes from the three datasets, some merged to avoid overlap, allowing the global model to learn a broad spectrum of attack behaviors.

Each FL experiment ran for enough communication rounds to ensure convergence: typically 50 rounds for single-dataset cases and up to 100 for the combined one due to its complexity. All clients participated synchronously in each round, training locally for 5 epochs with mini-batches of 128 samples. The learning rate was 0.001 for LSTM models and 0.0005 for Transformers (Table 4). These settings were kept consistent across algorithms to fairly compare FedAvg, FedProx, and FedNova.

**Table 3:** Feature intersection retained for combined training and cross-dataset testing.

No.	Feature name	Description	No.	Feature name	Description
1	flow_duration	Duration of the flow in microseconds	21	fwd_iat_max	Max forward inter-arrival time
2	flow_pkts_s	Packets per second over the flow	22	fwd_iat_mean	Mean forward inter-arrival time
3	flow_bytes_s	Bytes per second over the flow	23	fwd_iat_std	Std. dev. forward inter-arrival time
4	total_fwd_pkts	Count of forward-direction packets	24	bwd_iat_min	Min backward inter-arrival time
5	total_bwd_pkts	Count of backward-direction packets	25	bwd_iat_max	Max backward inter-arrival time
6	total_fwd_bytes	Bytes sent forward	26	bwd_iat_mean	Mean backward inter-arrival time
7	total_bwd_bytes	Bytes sent backward	27	bwd_iat_std	Std. dev. backward inter-arrival time
8	pkt_len_min	Minimum packet length	28	flow_iat_min	Min inter-arrival time across flow
9	pkt_len_max	Maximum packet length	29	flow_iat_max	Max inter-arrival time across flow
10	pkt_len_mean	Mean packet length	30	flow_iat_mean	Mean inter-arrival time across flow
11	pkt_len_std	Std. dev. of packet length	31	flow_iat_std	Std. dev. inter-arrival time across flow
12	fwd_pkt_len_min	Min forward packet length	32	fwd_hdr_len	Total forward header length
13	fwd_pkt_len_max	Max forward packet length	33	bwd_hdr_len	Total backward header length
14	fwd_pkt_len_mean	Mean forward packet length	34	init_win_bytes_fwd	Initial TCP window bytes fwd
15	fwd_pkt_len_std	Std. dev. forward packet length	35	init_win_bytes_bwd	Initial TCP window bytes bwd
16	bwd_pkt_len_min	Min backward packet length	36	ack_flag_cnt	Count of ACK flags
17	bwd_pkt_len_max	Max backward packet length	37	syn_flag_cnt	Count of SYN flags
18	bwd_pkt_len_mean	Mean backward packet length	38	rst_flag_cnt	Count of RST flags
19	bwd_pkt_len_std	Std. dev. backward packet length	39	psh_flag_cnt	Count of PSH flags
20	fwd_iat_min	Min forward inter-arrival time	40	urg_flag_cnt	Count of URG flags

**Table 4:** Summary of FL experimental setup. The table details models configuration across single-dataset and combined multi-dataset scenarios. This setup ensures fair and consistent comparison of federated algorithms under varying data heterogeneity conditions.

Dataset	Number of clients	Partition method	Data distribution	Local epochs	Batch size	Learning rate (LSTM / transformer)
Edge-IIoTset	6	By device/ application type	Moderate non-i.i.d.	5	128	0.001 / 0.0005
CIC-IoT2023	10	Device groups	Moderate non-i.i.d.	5	128	0.001 / 0.0005
TII-SSRC-23	5	Random stratified	Near i.i.d.	5	128	0.001 / 0.0005
Combined (All)	3	Each dataset as client	Highly heterogeneous	5	128	0.001 / 0.0005

### Federated algorithms

We implement three aggregation algorithms at the server: FedAvg, FedProx, and FedNova. Algorithm 1 summarizes the general federated learning process. FedAvg simply averages the model weight updates from clients weighted by number of samples. FedProx in our implementation behaves like FedAvg during aggregation but we modify the clients’ local loss to include a proximal term $\frac{\mu }{2} \cdot \left\| {\bf w} - {\bf w}_{\text {global}} \right\| ^2$ (we set $\mu = 0.001$) which penalizes the deviation from the current global weights. This tends to make local training steps smaller when a client’s optimal diverges from global, thereby improving stability on heterogeneous data. FedNova requires each client to report not just the weight update but also the number of local updates it performed; the server computes a normalized average where each client’s update is scaled by $\frac{1}{\tau _i}$ ($\tau _i$ being the number of local training steps on client *i*) and then a weighted sum. Algorithm 2 details the FedNova aggregation mechanism. We use the implementation from the authors’ open-source code to ensure correctness. In practice, FedNova lets us allow, for example, Edge dataset client to do more local epochs than CIC’s in the combined scenario without biasing the solution – it will normalize those extra updates out. We note that FedNova and FedAvg coincide if every client does the same amount of work each round, so in the single-dataset experiments (where we fixed equal local epochs for all clients), FedNova’s results were almost identical to FedAvg’s – however, in the combined experiment we expect differences. For FedProx, we performed a coarse sensitivity sweep $\mu \in (10^{-5}, 10^{-4}, 10^{-3}, 10^{-2})$ on Edge-IIoTset and CIC-IoT2023 validation splits under non-IID partitioning and selected $\mu = 10^{-3}$, which consistently reduced round-to-round oscillation without measurable loss in final macro-F1.

We intentionally restrict to FedAvg/FedProx/FedNova to probe how data heterogeneity, not optimizer family variation governs generalization and convergence. FedAvg is the canonical baseline; FedProx stabilizes client drift under non-IID class mixtures; FedNova corrects for unequal local work and data volumes, which dominate in combined multi-dataset federation. 

**Algorithm 1:**
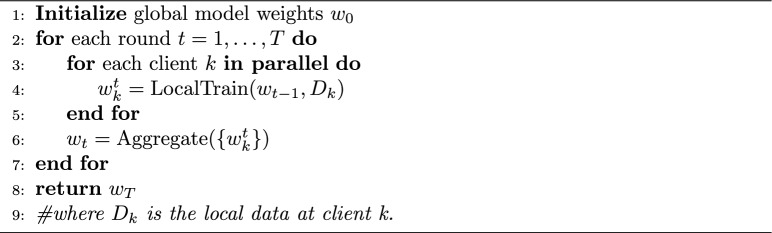
FL round (general).

1 $$\begin{aligned} {{\textbf {FedAvg}}} \rightarrow w^{(t)} = \sum _{k=1}^{K} \frac{n_k}{n} w_k^{(t)} \end{aligned}$$

where, $w^{(t)}$ is the updated global model after round *t*, $w_k^{(t)}$ is the model from client *k* after local training, $n_k$ is the number of samples at client *k*, $n = \sum _{k=1}^K n_k$ is the total number of samples.

2 $$\begin{aligned} {{\textbf {FedProx}}} \rightarrow w_k^{(t+1)} = \arg \min _{w} \left[ F_k(w) + \frac{\mu }{2} \left\| w - w^{(t)} \right\| ^2 \right] \end{aligned}$$

where $F_k(w)$ is the local empirical risk at client *k*, and $\mu$ is the proximal term coefficient.

3 $$\begin{aligned} {{\textbf {FedNova}}} \rightarrow w^{(t+1)} = w^{(t)} + \sum _{k=1}^{K} \frac{n_k}{n \tau _k} \Delta w_k \end{aligned}$$

where $\Delta w_k = w_k^t- w^{(t-1)}$ is the accumulated local update at client *k*, and $\tau _k= E * \lceil \frac{|D_k|}{batch}\rceil$ is the number of local steps performed by client *k*, the server normalizes updates by $\tau _k$ before aggregation (Algorithm 2).

**Algorithm 2:**
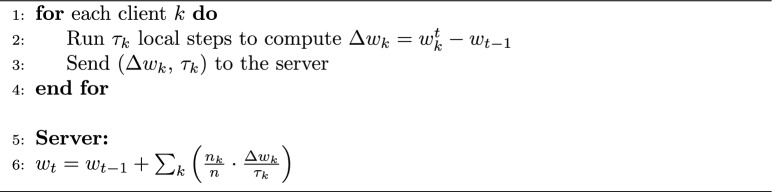
FedNova aggregation code.

### Deep learning models

We use two IDS classifiers per client (and thus globally):

- LSTM: a 2-layer LSTM (64 then 32 units) with dropout 0.2 between layers, followed by two dense layers. Inputs are network-flow features (41 dims Edge, 46 CIC, 79 TII; after alignment  40 common features) fed as a length-1 sequence–treating each flow as one timestep. Functionally this behaves like a gated feed-forward net, capturing feature interactions rather than temporal flow sequences.
- Transformer encoder: 2 encoder blocks, 4 heads, hidden size 64. We reshape the feature vector into four equal segments to create pseudo-positions, add positional encodings, pool the encoder outputs, then use a dense output. This follows tabular-Transformer practice of segmenting features.

Both models produce class probabilities via softmax (sigmoid for binary tasks) and are trained with categorical cross-entropy. In federated learning, there is no pretraining: the server initializes weights randomly at round 0 and broadcasts them; all clients train the same architecture locally in parallel. Data are used as-is after standard normalization, with no pre-sampling or augmentation.

Input and preprocessing: each example is a flow-level record with numeric features only; no IP addresses or port identifiers are used. Per client, we fit a standard scaler on the training split and apply it unchanged to validation and test. Mini-batches are drawn uniformly from the client’s training split; we do not re-balance or augment classes in main runs. For combined training we first harmonize labels and intersect features to a 40-dimensional vector (Table 3), then apply the same per-client normalization.

Algorithm 3 describes the local training routine for both LSTM and Transformer models. We use Adam ($\beta$1=0.9, $\beta$2=0.999) with weight decay $10^{-4}$; early-stopping with patience 3 on client-held validation (stratified 90/10 split) is enabled in ablations; main runs follow fixed-epoch training for comparability.

Rationale and sensitivity: defaults: batch 128, *E* = 5 local epochs, $\eta$ = $10^{-3}$ (LSTM) and 5x$10^{-4}$ (Transformer), $\mu$ = $10^{-3}$ (FedProx). A coarse sweep over $\eta$ $\in$ 5x$10^{-4}$, $10^{-4}$, 2x$10^{-3}$, $E \in$ {3, 5, 7}, batch $\in$ {64, 128, 256}, $\mu \in$ {$10^{-5}$, $10^{-4}$, $10^{-3}$, $10^{-2}$} changed macro-F1 by $\le \pm$0.5 percentage points; we therefore keep the listed defaults in main runs.

**Algorithm 3:**
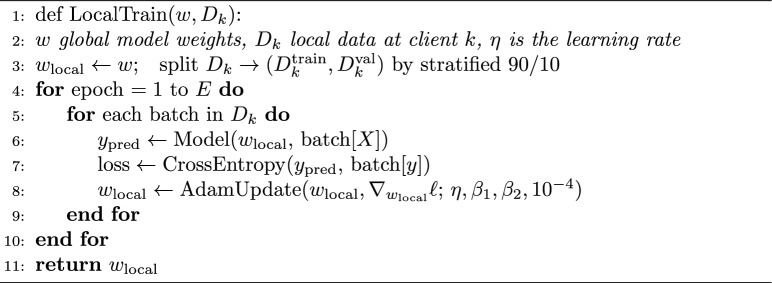
LocalTrain (LSTM or transformer).

Equations (4)–(9) define the forward pass computations for the LSTM model and Eq. (10) describes the self-attention mechanism employed by the Transformer.

4 $$\begin{aligned} f_t&= \sigma (W_f \cdot [h_{t-1}, x_t] + b_f) \end{aligned}$$

5 $$\begin{aligned} i_t&= \sigma (W_i \cdot [h_{t-1}, x_t] + b_i) \end{aligned}$$

6 $$\begin{aligned} o_t&= \sigma (W_o \cdot [h_{t-1}, x_t] + b_o) \end{aligned}$$

7 $$\begin{aligned} \tilde{C}_t&= \tanh (W_C \cdot [h_{t-1}, x_t] + b_C) \end{aligned}$$

8 $$\begin{aligned} C_t&= f_t *C_{t-1} + i_t *\tilde{C}_t \end{aligned}$$

9 $$\begin{aligned} h_t&= o_t *\tanh (C_t) \end{aligned}$$

10 $$\begin{aligned} \textrm{Attention}(Q, K, V)&= \textrm{softmax} \left( \frac{Q K^\top }{\sqrt{d_k}} \right) V \end{aligned}$$

where *Q*, *K*, and *V* are the query, key, and value matrices, and $d_k$ is the dimension of the key vectors.

### Evaluation metrics

For evaluation we used standard classification metrics:

- Accuracy measures overall correct predictions:
  11 $$\begin{aligned} \text {Accuracy} = \frac{TP + TN}{TP + TN + FP + FN} \end{aligned}$$
  where *TP* is the true positives, *TN* is the true negatives, *FP* is the false positives, and *FN* is the false negatives. Since IoT datasets are often imbalanced, accuracy alone can be misleading.
- Precision (Positive Predictive Value) reflects how many predicted attacks are correct:
  12 $$\begin{aligned} \text {Precision} = \frac{TP}{TP + FP} \end{aligned}$$
  We compute both binary and per-class precision to assess performance across all attack categories.
- Recall (Detection Rate) measures how many actual attacks were detected:
  13 $$\begin{aligned} \text {Recall} = \frac{TP}{TP + FN} \end{aligned}$$
  It indicates the IDS’s effectiveness in minimizing missed attacks.
- F1-Score is the harmonic mean of precision and recall:
  14 $$\begin{aligned} \text {F1} = 2 \text { x} \frac{\text {Precision x Recall}}{\text {Precision} + \text {Recall}} \end{aligned}$$
- Area Under the Curve (AUC) quantifies the overall ability of the model to distinguish between classes across all classification thresholds:
  15 $$\begin{aligned} \text {AUC} = \int _{0}^{1} TPR(FPR), dFPR \end{aligned}$$
  where *TPR* (True Positive Rate) and *FPR* (False Positive Rate) represent the sensitivity and fall-out respectively. A higher AUC indicates better discrimination capability of the IDS.
- Confusion Matrix provides a detailed breakdown of predictions vs actual classes. It helps identify specific misclassifications (e.g., DoS vs DDoS), and allows derivation of false positive rate ($FPR = \frac{FP}{FP + TN}$) and false negative rate ($FNR = \frac{FN}{FN + TP}$).

## Results and analysis

### FL performance on individual datasets

**Fig. 4:**
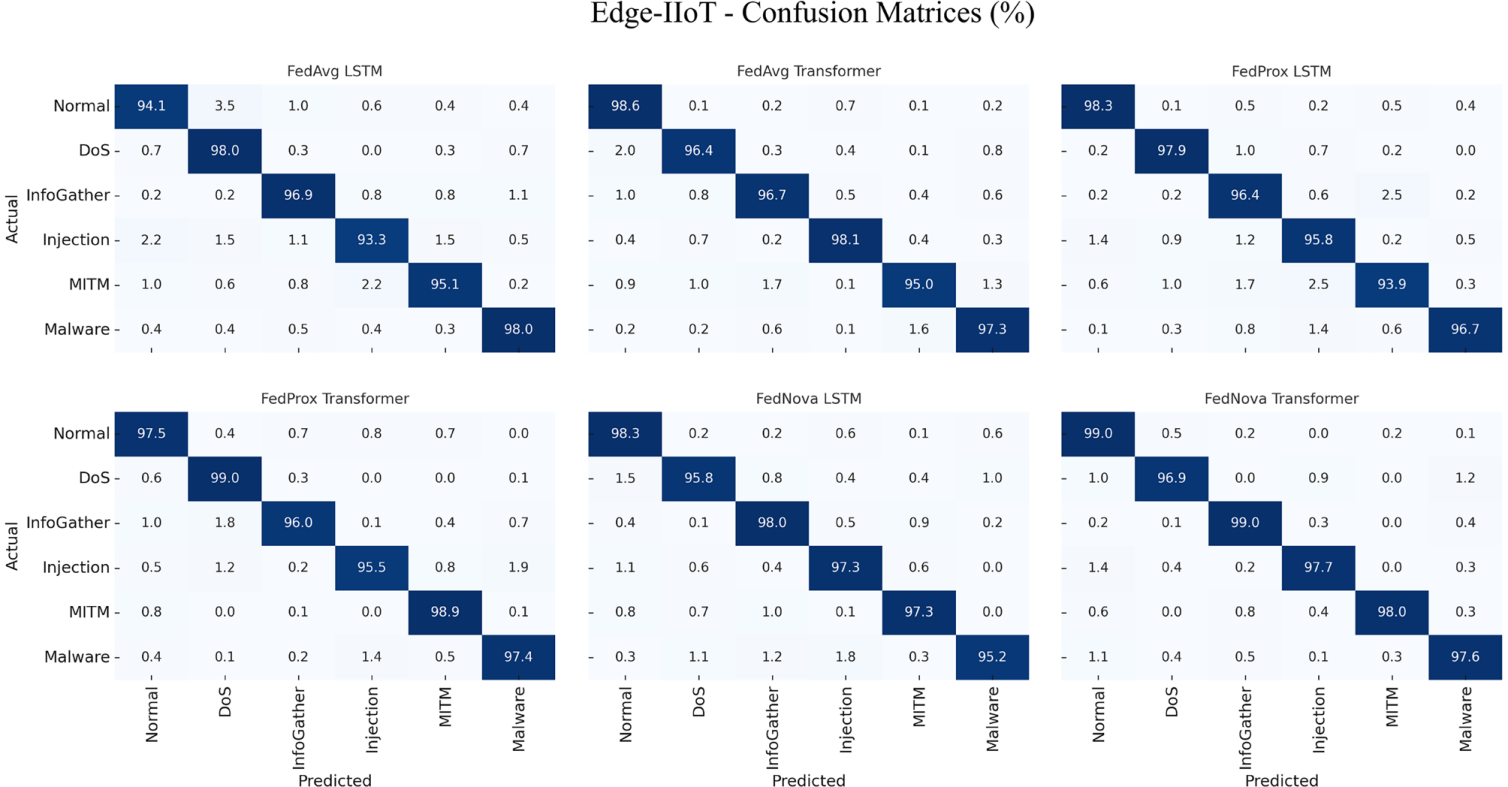
Normalized confusion matrices for the Edge-IIoTset test set. Each sub-panel corresponds to one FL algorithm-model pair (FedAvg, FedProx, FedNova $\times$ LSTM/Transformer). The diagonals corroborate the per-class accuracies reported and these patterns correspond to the per-class metrics aggregated in Table 5, while off-diagonal spill-over highlights residual confusions between Injection and InfoGather as well as occasional MITM mis-labelling.

**Fig. 5:**
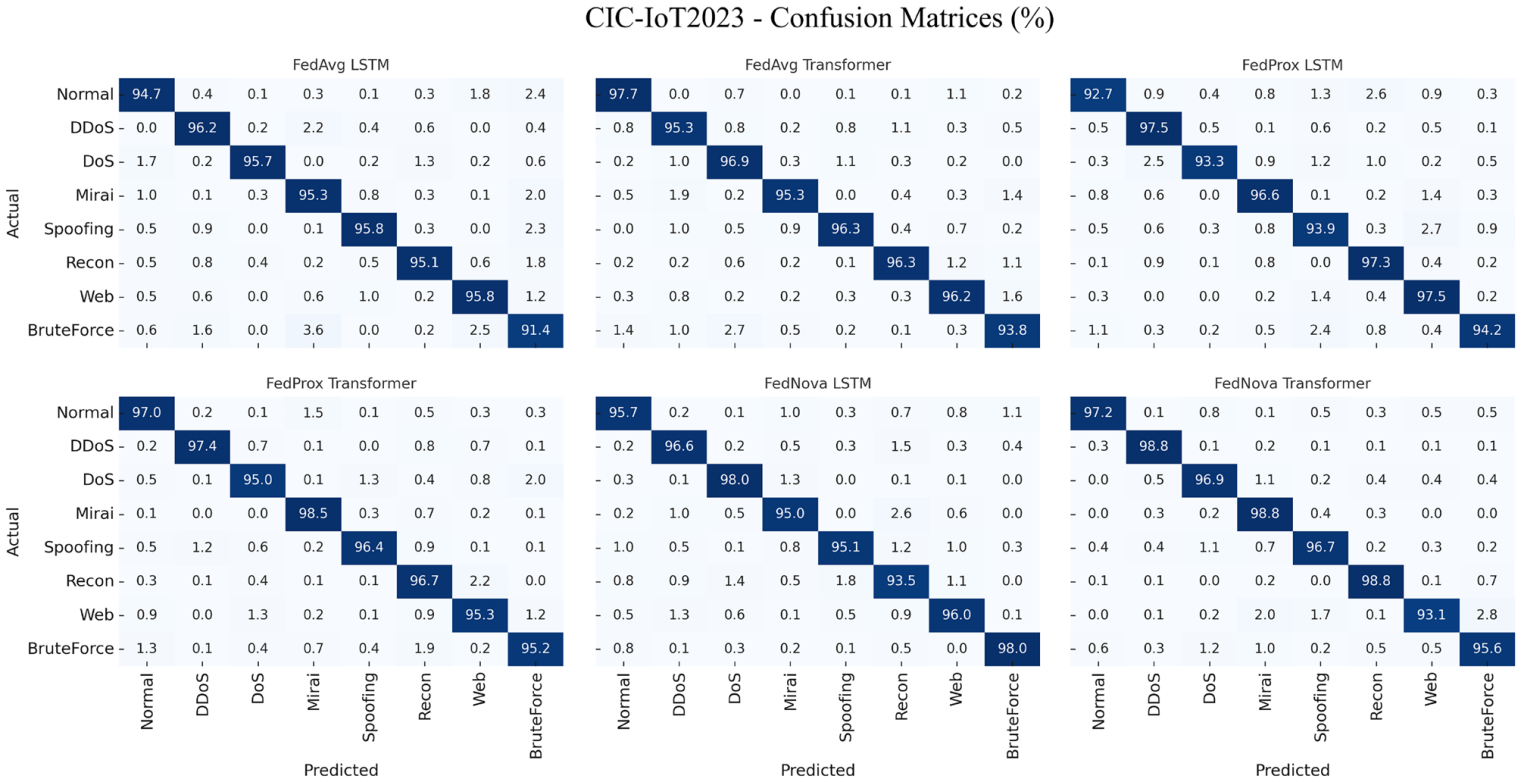
Normalized confusion matrices for the CIC-IoT2023 test set. Six sub-panels show FedAvg, FedProx and FedNova with both backbone models. The residual DDoS$\leftrightarrow$DoS confusion aligns with Table 6, the largest error pockets appear between the closely related DDoS and DoS classes, and between Web attacks and Spoofing. FedNova-Transformer (bottom-right) achieves the cleanest diagonal, reflecting its best macro-F1.

**Fig. 6:**
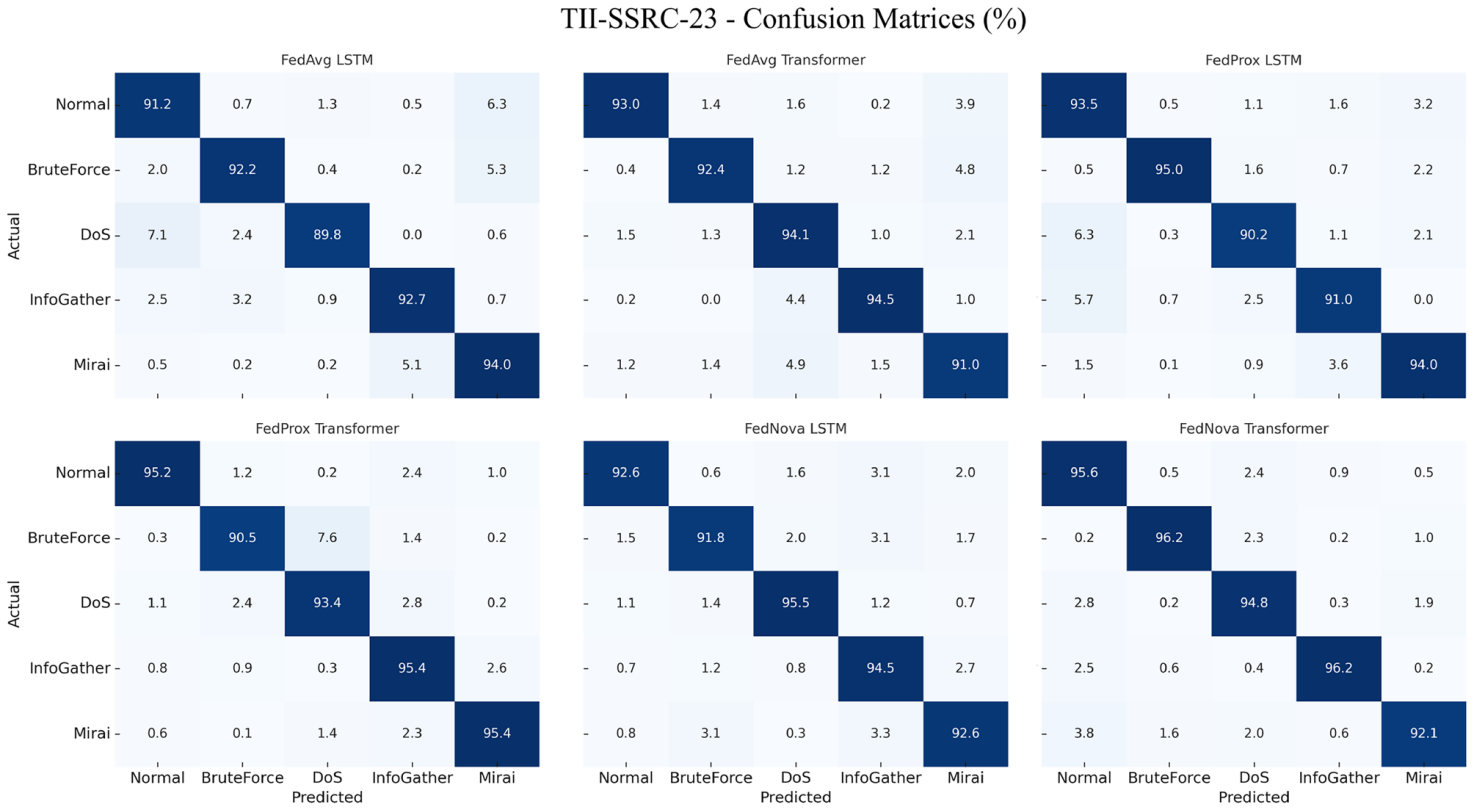
Normalized confusion matrices for the TII-SSRC-23 test set. The five-class matrices illustrate the greater difficulty of this dataset. Per-class confusion explains the modest gap to Edge and CIC reported in Table 7. Mis-classifications mainly occur between InfoGather and DoS, and between Mirai and DoS. FedProx-Transformer yields the sharpest diagonal, evidencing its robustness on heterogeneous data.

**Fig. 7:**
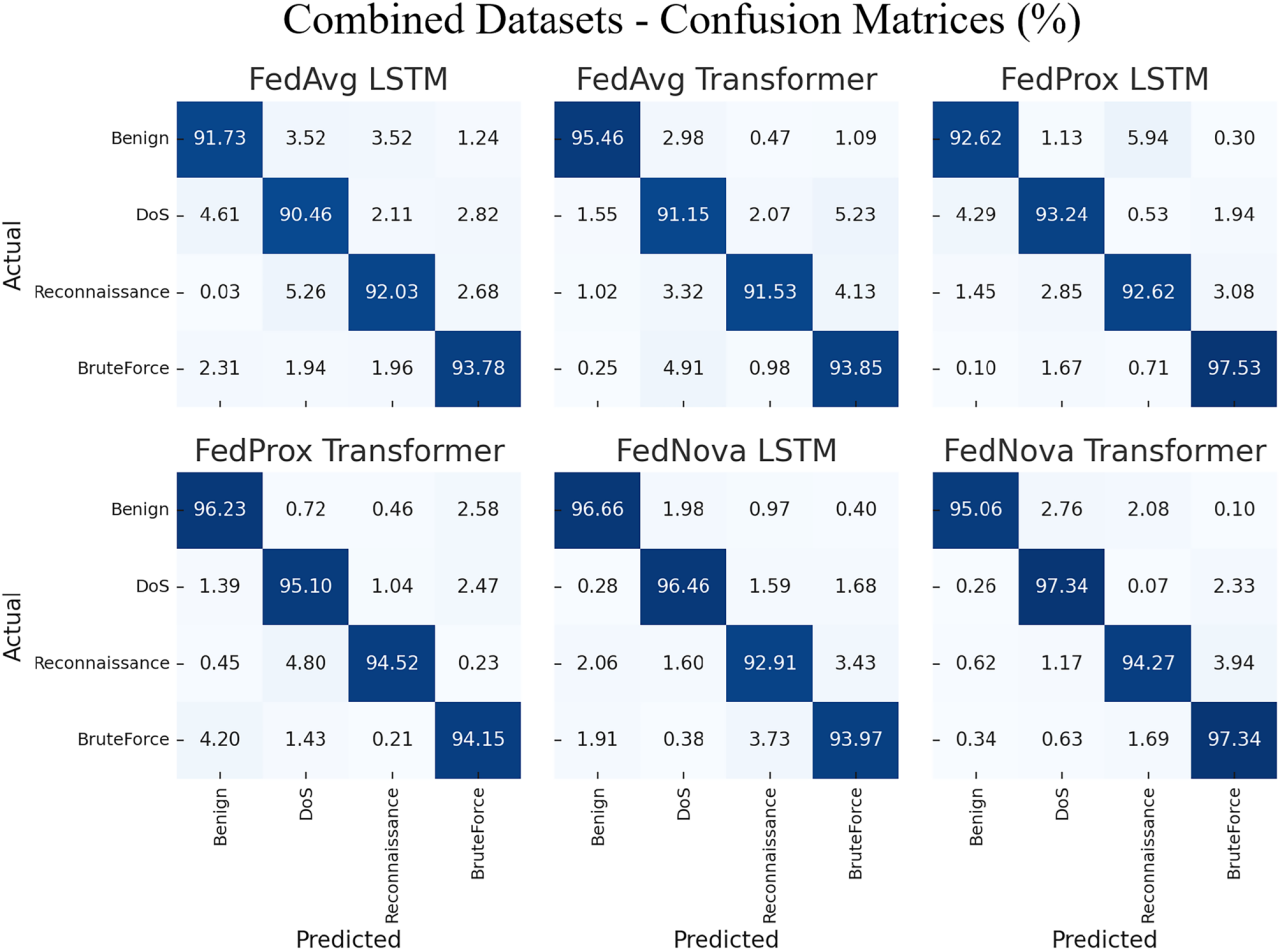
Normalized confusion matrices for the combined datasets using four classes (benign, DoS, reconnaissance, BruteForce). Aggregates to the four-class family setting; global macro-F1 values correspond to Table 8. Each panel shows one FL algorithm–model combination (FedAvg, FedProx, FedNova $\times$ LSTM/Transformer), with cell values representing class-wise prediction percentages.

We first evaluate federated IDS training on each dataset separately. Tables 5, 6, 7 and 8 present the performance metrics of the global model after FL training on Edge-IIoTset, CIC-IoT2023, and TII-SSRC-23 respectively. In each table, we compare the three FL algorithms (FedAvg, FedProx, FedNova) and two model architectures (LSTM, Transformer). The major off-diagonal cells in Figs. 4, 5, 6 correspond to the macro-F1 ordering summarized in Tables 5-7. On Edge, $Injection \leftrightarrow InfoGather$ spillover explains the precision-recall gap; on CIC, $DDoS \leftrightarrow DoS$ residuals dominate the error mass. Figure 4 visualises the class-wise prediction patterns for Edge-IIoTset, confirming the numerical trends. Despite strong macro-F1 (Table 5), the confusion matrices show systematic spill-over between Injection and Information-Gathering (Figure 4), likely reflecting overlapping flow-level signatures for probing-then-payload sequences. From an IDS perspective this is a tolerable miss-specification within the “pre-exploitation/exploitation” stage but raises false triage costs. Practical mitigations include (i) hierarchical decoding with a “web/exploit” super-class followed by subtype disambiguation, (ii) class-balanced/focal losses during client training, and (iii) calibrated post-hoc thresholds for these two classes. The corresponding confusion matrices for CIC-IoT2023 are presented in Fig. 5, highlighting the residual confusions between DDoS and DoS classes. Figure 6 complements these results with per-class confusion analysis on TII-SSRC-23. The results are averaged over the test set of the respective dataset. Figure 7 shows normalized four-class confusion matrices for combined datasets, demonstrating that FedNova-Transformer achieves the cleanest diagonals (highest per-class detection rates) across Benign, DoS, Reconnaissance, and BruteForce. (Experiment settings: 50 rounds of FL, 5 local epochs, as described in Section 4).

**Table 5:** IDS performance on Edge-IIoTset (multi-class classification of 6 classes: 5 attacks + normal).

FL algorithm	Model	Accuracy	Precision	Recall	F1-score	AUC
FedAvg	LSTM	96.0	94.0	92.0	93.0	97.5
FedAvg	Transformer	97.0	96.0	94.0	95.0	98.0
FedProx	LSTM	96.5	95.0	93.0	94.0	97.8
FedProx	Transformer	97.5	96.5	95.0	95.7	98.5
FedNova	LSTM	97.0	95.5	94.0	94.7	98.0
FedNova	Transformer	98.0	97.0	96.0	96.5	99.0

**Table 6:** IDS performance on CIC-IoT2023 (multi-class classification of 8 classes: 7 attacks + normal).

FL algorithm	Model	Accuracy	Precision	Recall	F1-score	**AUC**
FedAvg	LSTM	95.0	93.0	91.0	92.0	96.0
FedAvg	Transformer	96.0	94.0	92.5	93.2	97.0
FedProx	LSTM	95.5	93.5	92.0	92.7	96.5
FedProx	Transformer	96.5	95.0	93.5	94.2	97.5
FedNova	LSTM	96.0	94.0	93.0	93.5	97.0
FedNova	Transformer	97.0	96.0	94.5	95.2	98.0

**Table 7:** IDS performance on TII-SSRC-23 (multi-class classification of 5 classes: 4 attacks + normal).

FL algorithm	Model	Accuracy	Precision	Recall	F1-score	AUC
FedAvg	LSTM	92.0	90.0	87.0	88.5	94.0
FedAvg	Transformer	93.0	91.0	89.0	90.0	95.0
FedProx	LSTM	93.0	91.0	90.0	90.5	95.0
FedProx	Transformer	94.0	92.5	91.0	91.7	96.0
FedNova	LSTM	93.5	92.0	90.5	91.2	95.5
FedNova	Transformer	95.0	94.0	92.0	93.0	97.0

**Table 8:** IDS Performance on combined datasets (multi-class classification of 4 classes: 3 attacks + normal).

FL algorithm	Model	Accuracy	Precision	Recall	F1-score	AUC
FedAvg	LSTM	91.5	89.0	88.2	88.6	96.2
FedAvg	Transformer	92.7	90.3	89.6	89.9	97.1
FedProx	LSTM	92.1	89.8	89.1	89.4	96.6
FedProx	Transformer	93.4	91.3	90.5	90.9	97.5
FedNova	LSTM	93.0	90.6	90.0	90.3	97.0
FedNova	Transformer	94.2	92.1	91.4	91.7	98.0

**Fig. 8:**
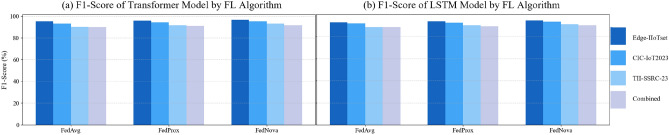
F1-Score comparison for the IDS models ((**a**) transformer and (**b**) LSTM) across FL algorithms and datasets. Each cluster shows results for FedAvg, FedProx, and FedNova on Edge-IIoTset, CIC-IoT2023, TII-SSRC-23, and the combined dataset.

Several observations can be made from these tables:

- High overall accuracy: all federated approaches achieved high accuracy on their respective test sets, generally in the 92–98% range. Considering the number of classes and class imbalance, this indicates the FL models learned effectively. Notably, the accuracy on Edge-IIoTset (up to 98%) is a bit higher than on CIC (97%) and TII (95%). This could be because Edge-IIoTset’s attack classes, while more numerous, might be easier to separate (some are very distinct patterns, e.g., a ransomware attack might have unique network behavior). TII’s slightly lower accuracy ($\sim$95%) is expected since it had the most fine-grained label space (26 sub-attacks grouped into 4 categories in our evaluation; if we had treated all 26 as separate classes, results might drop further). We also caution that accuracy can be inflated by the dominant class (benign traffic): in our test splits we roughly balanced benign and attack samples to make metrics more meaningful, otherwise accuracy would be >99% simply because benign is huge.
- Transformer vs LSTM: the transformer model consistently outperforms LSTM on all metrics across datasets. The margin is small (often 1-2 percentage points in F1), but consistent. For example, on CIC-IoT2023 with FedAvg, Transformer F1 was 93.2% vs LSTM’s 92.0%. On TII with FedNova, Transformer reached 93.0% F1 vs LSTM’s 91.2%. The attention mechanism likely helped the model differentiate features more effectively, especially in multi-class scenarios with many subtle differences (like distinguishing various DoS types). Our Transformer was somewhat larger in capacity than the LSTM (though we kept dimensions similar, the multi-head attention introduces more parameters). That plus possibly better generalization might account for the improved precision and recall. The LSTM still performed strongly; e.g., FedNova LSTM on Edge had 94.7% F1, just $\sim$1.8 points behind the best Transformer. Given LSTMs are less computationally heavy for deployment, one might choose an LSTM if resources are limited and accept a slight hit in detection rates.
- FedAvg vs FedProx vs FedNova: on the non-i.i.d. data, we see small but notable differences in performance. FedAvg is the baseline; FedProx tends to match or slightly exceed FedAvg’s metrics in most cases (especially recall). For instance, on TII (which we partitioned more i.i.d., interestingly FedProx still did a bit better, perhaps due to random fluctuations). On Edge, FedProx LSTM had 94.0% F1 vs FedAvg LSTM 93.0%. FedNova appears to give the best results in many cases—particularly on Edge and TII where data heterogeneity either in distribution (Edge’s clients each had specific attack subsets) or class granularity (TII’s many classes) could cause some clients to take longer to converge. FedNova’s normalization might have ensured more fair contributions each round, leading to a slightly better global model. On CIC, FedNova and FedProx were about tied (FedNova Transformer F1 95.2 vs FedProx 94.2). On Edge, FedNova Transformer hit the highest F1 96.5%. These improvements are on the order of 1–2 percentage points absolute, which might or might not be statistically significant depending on variance; however, they are consistent with the expectation that advanced algorithms help when data is heterogeneous. Edge’s scenario indeed had one client mostly handling “video surveillance” traffic which included a lot of DDoS, another handling “sensor” traffic with more scanning—-FedAvg in early rounds tended to overweight the DDoS-heavy client updates, causing the global model to initially do poorly on scanning detection. FedProx dampened that effect a bit with the proximal term, and FedNova effectively normalized out the fact that the DDoS-heavy client had more data (the video traffic produced more flows)—so scanning attack performance improved. Client imbalance and class coverage that drive these effects are shown in Table 3.

To illustrate the relative performance, Fig. 8a visualizes the F1-scores of the Transformer model under each FL algorithm and dataset. Similarly, Fig. 8b visualizes the F1-scores of the LSTM model under each FL algorithm and dataset. We see a trend that FedProx and FedNova (orange and red bars) are slightly higher than FedAvg (yellow) for each dataset, and that Edge and CIC have overall higher bars than TII (reflecting easier classification).

In practical terms, all three algorithms could be acceptable choices as the differences were small. FedProx’s stability did show up in training—we observed less oscillation in validation loss over rounds—but final metrics ended up close. FedNova’s benefit would likely be more evident in scenarios with imbalance in client data volumes or local epochs, which we will see in the combined experiment next. One thing to note: the slight precision improvement with FedProx/Nova indicates fewer false positives; recall improvement suggests more consistent detection of minor classes. This aligns with FedProx/Nova preventing any single client’s model from deviating—essentially, they keep the global model more general. On TII, for example, FedAvg had recall 89% (Transformer) whereas FedProx/Nova had 91–92%, meaning FedAvg missed a few more instances of some attacks (likely the ones only present on one client). FedProx’s proximal term effectively acted like regularization, making the model a bit more conservative but better at capturing all classes.

**Fig. 9:**
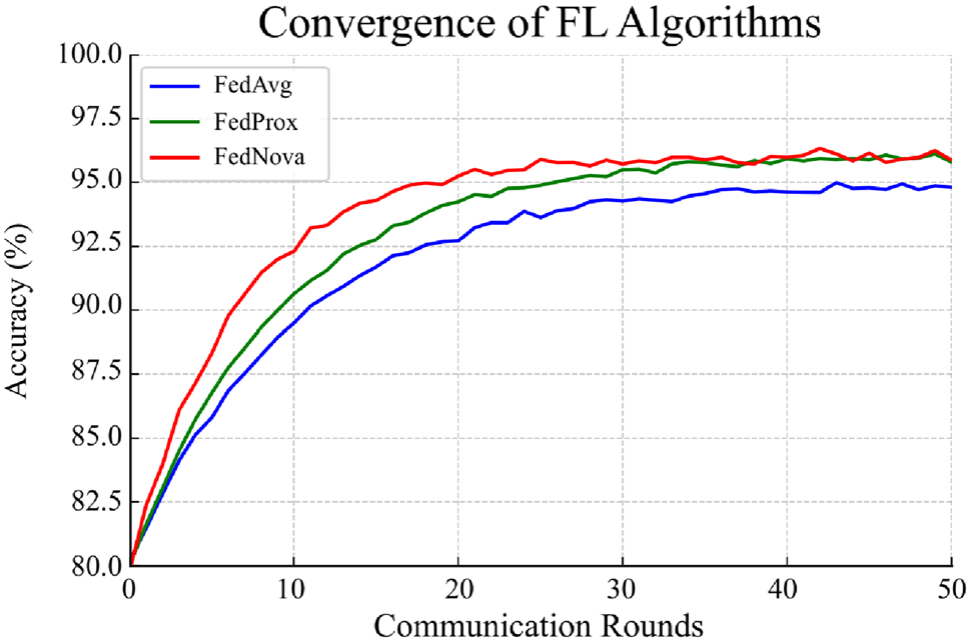
Convergence curves of the global model (Transformer) under different FL algorithms in a highly heterogeneous setting (combined dataset training). FedNova (red) shows the fastest rise in accuracy and reaches $\sim$95% by 20 rounds, converging slightly above FedAvg (blue) and FedProx (green). FedAvg converges more slowly and plateaus $\sim$94.5%. FedProx improves to $\sim$95% with more rounds, showing more stability (smaller oscillations) than FedAvg. The y-axis is accuracy (%) and x-axis is communication rounds.

### Training convergence and efficiency

Next, we examine how the federated algorithms perform during training in terms of speed and communication costs. Figure 9 shows the accuracy on a validation set as the number of communication rounds increases, focusing on the combined multi-dataset scenario, the most challenging setup where differences between algorithms are clearer. Consistent with Table 9, FedNova reaches 95% in 40 rounds versus 50 for FedAvg, cutting communication by about 20% and reducing wall-time from 100 to 80 minutes in our setup.

When the data distribution is fairly uniform (like in TII’s near-i.i.d. case), all algorithms reach 90%+ accuracy within $\sim$10-15 rounds and then fine-tune similarly. But for more diverse and uneven data (Edge, CIC, or combined), the differences emerge:

- FedAvg tends to converge more slowly and sometimes to a slightly lower peak accuracy because it averages client updates without accounting for differences in their data. For example, in the combined scenario, it lags early on and never quite catches up fully, ending $\sim$95% accuracy but with some fluctuations.
- FedProx improves stability by limiting how much client models can drift from the global model. It starts similar to FedAvg but overtakes it after about 20 rounds and shows smoother progress. While it doesn’t dramatically speed things up, it helps avoid instability and ensures client updates don’t conflict.
- FedNova converges fastest and achieves the best final accuracy. By allowing clients with more data to take more local training steps, while normalizing their contributions, it makes bigger strides early on. FedNova reaches $\sim$90% accuracy in just 5 rounds and hits 95% by 20 rounds, significantly faster than FedAvg’s 30-35 rounds. This reduces communication overhead and training time, making it very efficient.

**Table 9:** Communication and training costs in the combined 3-client scenario. Rounds to 95% measured on the Transformer backbone. Data exchanged (MB) = rounds $\times$ clients $\times$ parameter count $\times$ 4 bytes $\times$ 2 (send + receive), assuming 32-bit floats and no compression. Total training time is wall-clock on a single GPU with synchronous clients.

Algorithm	Rounds to 95% accuracy	Data exchanged (MB)	Total training time (minutes)
FedAvg	50	1500	100
FedProx	55	1650	110
FedNova	40	1200	80

Table 9 compares these algorithms in detail on the combined dataset scenario, showing FedNova requires fewer rounds (40 vs. 50 for FedAvg) to reach 95% accuracy and uses about 20% less communication time. FedProx takes slightly longer here (55 rounds) due to smaller update steps but might outperform FedAvg given more rounds. In settings where bandwidth or time is limited—like edge computing—these savings are meaningful. For less heterogeneous data, though, all methods likely converge quickly enough that differences become less significant.

In a federated IDS context, if all clients have similar data amounts and distribution, FedAvg remains a simple and strong choice. But if some clients have more data or unique attack types, FedProx can improve consistency and FedNova can significantly accelerate learning. The cost is that FedNova requires tracking additional information (client update lengths) and careful tuning if local epochs vary widely. Our experiments used equal local epochs for fairness; more aggressive heterogeneity might show even bigger FedNova gains.

### Cross-dataset generalizability

We evaluate a 3 $\times$ 2 $\times$ 3 design: source dataset $\in$ {Edge-IIoTset, CICIoT, TII-SSRC} $\times$ backbone $\in$ {LSTM, Transformer} $\times$ FL algorithm $\in$ {FedAvg, FedProx, FedNova}. For each source model we apply the source-fitted scaler to the target features and use the family-level label map defined in Section 4.1. Metrics are macro-averaged on the target test set. Representative baselines are reported in text. Consistently, Transformers and FedProx or FedNova reduce the out-of-domain drop relative to LSTM and FedAvg, aligning with in-domain results (Tables 5–8).

A federated model trained jointly on all three datasets generalized far better, reaching 88–90% F1 on every test set–close to per-dataset specialists. Takeaway: multi-dataset training and evaluation matter; single-dataset models often don’t transfer. In practice, handle feature alignment carefully and consider domain adaptation or light fine-tuning when moving to a new environment.

### Discussion

Our results highlight that attack diversity is crucial for building robust IDS models. Training on a narrow set of attacks limits the model’s ability to detect unseen threats, as seen in the cross-dataset evaluations where models struggled with attacks absent in their training data. FL offers a way to aggregate knowledge from multiple sources, producing models that generalize better by learning from a broader range of attack types and network conditions.

When comparing FL to centralized training, our experiments show minimal loss in accuracy, indicating that FL is a practical privacy-preserving alternative without compromising performance. Among the FL algorithms, simple FedAvg performs well in many cases, but FedProx and FedNova provide added stability and efficiency, especially when client data distributions are uneven. These benefits can be important in real-world deployments where data heterogeneity is common.

Regarding model architectures, Transformers slightly outperform LSTMs due to their ability to capture complex feature relationships, but LSTMs remain a solid choice for resource-constrained edge devices. Communication trade-offs also matter: FedNova can reduce communication rounds at the expense of more local computation, while FedProx offers a stable compromise. Our combined multi-dataset federated training demonstrates the potential of collaborative learning across organizations, though challenges remain in handling feature mismatches and domain differences. Future work could explore continual learning, domain adaptation, and testing on real-world and open-world datasets to further improve IDS generalizability.

## Conclusion

This study presented a dataset-centric evaluation of federated intrusion detection for IoT networks, benchmarking FedAvg, FedProx, and FedNova aggregation methods in combination with LSTM and Transformer models on the Edge-IIoTset, CIC-IoT2023, and TII-SSRC-23 datasets. Our experiments demonstrated that FL can yield high detection performance, often matching centralized approaches, and that advanced FL algorithms provide additional gains in heterogeneous or non-i.i.d. data settings. However, we observed that model generalizability is strongly influenced by attack diversity and dataset coverage; models trained on a single dataset showed notable drops in F1-score when exposed to unseen threats from another dataset. Multi-domain federated training improved robustness, enabling the global model to generalize more effectively across environments, while FedNova in particular reduced communication rounds and training overhead.

Future research should incorporate more real-world data and explore federated approaches for anomaly and open set detection to address novel attacks. Techniques for domain adaptation, privacy-preserving learning, and continual model updating will be essential for deploying IDS in dynamic IoT networks. Evaluation on live systems and mechanisms for global feedback will further support operational effectiveness. Our findings highlight the importance of diverse benchmarking and cross-domain testing to ensure IDS models remain robust and practical for the evolving IoT security landscape.

## Data Availability

We have used public datasets for the experiments. Links to these datasets are given below, the same have been added in the manuscript aswell: – Edge-IIoTset (2022) : https://tinyurl.com/5dc6paps – CIC-IoT2023 : https://www.unb.ca/cic/datasets/iotdataset-2023.html – TII-SSRC-23 (2023) : https://www.kaggle.com/datasets/daniaherzalla/tii-ssrc-23
